# Chemosensory-Related Genes in Marine Copepods

**DOI:** 10.3390/md20110681

**Published:** 2022-10-29

**Authors:** Vittoria Roncalli, Marco Uttieri, Iole Di Capua, Chiara Lauritano, Ylenia Carotenuto

**Affiliations:** 1 Integrative Marine Ecology Department, Stazione Zoologica Anton Dohrn, Villa Comunale, 80121 Napoli, Italy; 2 NBFC, National Biodiversity Future Center, 90133 Palermo, Italy; 3 Research Infrastructures for Marine Biological Resources Department (RIMAR)-Marine Organism Taxonomy Core Facility (MOTax), Stazione Zoologica Anton Dohrn, Villa Comunale, 80121 Napoli, Italy; 4 Ecosustainable Marine Biotechnology Department, Stazione Zoologica Anton Dohrn, Via Acton 55, 80133 Napoli, Italy

**Keywords:** copepods, transcriptome, gene discovery, ionotropic receptors, chemosensory proteins, gustatory receptors, odorant receptors

## Abstract

Living organisms deeply rely on the acquisition of chemical signals in any aspect of their life, from searching for food, mating and defending themselves from stressors. Copepods, the most abundant and ubiquitous metazoans on Earth, possess diversified and highly specified chemoreceptive structures along their body. The detection of chemical stimuli activates specific pathways, although this process has so far been analyzed only on a relatively limited number of species. Here, in silico mining of 18 publicly available transcriptomes is performed to delve into the copepod chemosensory genes, improving current knowledge on the diversity of this multigene family and on possible physiological mechanisms involved in the detection and analysis of chemical cues. Our study identifies the presence of ionotropic receptors, chemosensory proteins and gustatory receptors in copepods belonging to the Calanoida, Cyclopoida and Harpacticoida orders. We also confirm the absence in these copepods of odorant receptors and odorant-binding proteins agreeing with their insect specificity. Copepods have evolved several mechanisms to survive in the harsh marine environment such as producing proteins to respond to external stimulii. Overall, the results of our study open new possibilities for the use of the chemosensory genes as biomarkers in chemical ecology studies on copepods and possibly also in other marine holozooplankters.

## 1. Introduction

Sensing environmental cues, which inform the organisms of resources and risks, is used to obtain information on location, shelter, food (presence and quality), mates and predators [1]. Chemosensation has been observed in a broad range of taxa from bacteria to humans [2]. Among invertebrates, much information on how organisms perceive chemical cues is available for insects, in particular, in the fruit fly *Drosophila melanogaster* [1,3]. In these terrestrial animals, the detection of volatile (low-molecular-weight odorants and pheromones) and nonvolatile (tastants) chemicals occurs through olfactory and gustatory sensory structures called sensilla. These are usually localized in the antenna and mouthparts, but also in legs, wings and genitals [4]. Sensory neurons contained in the sensilla express different chemosensory receptor proteins that are able to perceive several chemical cues. In *D. melanogaster*, chemoreception is mediated by three multigene families: gustatory receptors (GRs), odorant receptors (ORs) and ionotropic receptors (IRs). GRs and ORs are transmembrane receptors [5,6]. IRs are a group of transmembrane ion channels evolved from the highly conserved ionotropic glutamate receptors (iGluR) present in all Protostomia but not in the Deuterostomia [7,8,9]. IRs are considered olfactory receptors detecting persistent and proximal cues, whereas ORs are fast-adapting receptors perceiving weak and distant chemicals [10].

In insects, based on their expression and localization, GRs are considered involved in the perception of taste and few odorants, but also in the detection of CO_2_ [11], whereas ORs detect pheromones [12] and a wide panel of different low-molecular-weight odorant molecules [13]. IRs can bind to both tastants and odorants, especially acids and amines, but are also involved in temperature perception and circadian rhythm control [14]. These receptors often require expression of co-receptors (e.g., IR8a, IR25a and IR76b), which is necessary for the functioning of the receptor channels [10]. IRs have been classified into two groups: conserved “antennal IRs” and “divergent IRs”, which are species-specific and do not have known homologues [15]. Conserved IRs include many classes, of which IR8a, IR21a, IR25a, IR76a and IR93a have been better investigated. Of those, IR8a, IR25a, IR76b, and IR93a are “co-receptor IRs” because they are coexpressed with other IRs in cells and necessary for the function of the receptor channels [16]. The chemosensory molecular process starts with the transport of the chemical cue (ligand) to the chemosensory receptor, via chemosensory binding proteins (CSPs) and insect-type odorant-binding proteins (OBPs) [17], which then activates a downstream signal transduction pathway leading to the organismal response [18]. GRs, ORs, IRs, CSPs and OBPs can be collectively called chemosensory-related genes (CRGs) [2]. 

In contrast to terrestrial environments, aquatic chemical ecology defined as the chemically mediated interaction between organisms and their environment, has a more recent history [19,20]. The classic view of chemosensory perception in the aquatic realm stated that all chemical cues are waterborne hydrophilic molecules [21]. However, the most recent literature supports the view that insoluble volatile and lipophilic chemical cues are also detected by aquatic organisms [22]. In particular, chemical cues produced and detected by aquatic organisms can be grouped according to their water solubility and volatility: volatile/insoluble, volatile/soluble, nonvolatile/soluble, and nonvolatile/insoluble [22]. Several marine terpenoids, e.g., methyl farnesoate and sesquiterpene, belong to volatile/insoluble molecules, and are involved in larval development [23] and food palatability [24]. Similarly, the volatile and water-soluble dimethylsulfoniopropionate (DMSP) acts as a food-finding cue in copepods [25], whereas other compounds belonging to this category are involved in host detection by the copepod parasite *Lepeophtheirus salmonis* [26]. Nonvolatile/soluble molecules such as odorless alkaloids, amino acids and nucleosides, play multiple roles in the aquatic environment, from regulation of mating, reproduction and defense [21].

Aquatic compounds present chemical structures calibrated to work both in contact (tastants) and distance (odorants) interactions, with the occurrence of some specific features distinguishing freshwater and marine compounds [20]. These can work over long distances, creating time-persistent gradients depending on fluid kinematic properties [27]. With reference to crustaceans, the vast majority of the work on the chemosensory system has been performed on Decapoda, while more limited knowledge is available for Copepoda [15,16,28]. Copepods, which are likely the most abundant metazoans on Earth, have successfully colonized every water environment and are crucial in the functioning of marine ecosystems [29]. In addition, their absolute and relative success is similar to that of insects [30] thanks to their phylogenetic age, speciosity and size [31]. As copepods rely on chemical signals for predator avoidance, prey searching and mate finding [28], deepening the comprehension of their chemical ecology is fundamental to understand the modalities by which different genes/enzymatic pathways are regulated.

In a recent study, the presence of IRs, GRs and CSPs has been examined in several copepod genomes and transcriptomes (publicly available on National Center for Biotechnology Information, NCBI) with the aim of establishing their evolution in Arthropoda [2]. CRGs were searched in the genome and transcriptome of *Eurytemora affinis*, in the genome of *Calanus finmarchicus* and in the transcriptomes of *Acartia fossae, Calanus sinicus, Caligus rogercresseyi*, *Lepeophtheirus salmonis, Lernaeidae cyprinacea*, *Mesocyclops edax* and *Tigriopus californicus*. The CRG diversity and distribution were then compared with those of insects. However, as the number of high-quality publicly available copepod transcriptomes has increased since [2], a powerful opportunity to better investigate the chemosensory pathway in those organisms is now open. Thus, the goal of our study has been to expand the identification of transcripts encoding for chemoreceptor proteins in copepods, and to compare them to homologs chemoreceptor proteins in *E. affinis,* in the insect *D. melanogaster* and the cladoceran *Daphnia pulex* [2]. We also reported relative expression of the identified CRGs across different developmental stages in *C. finmarchicus* and in response to toxic algae in both *C. finmarchicus* and *C. helgolandicus*. Our results shed light on the diversity and functioning of CRGs in key copepods species belonging to Calanoida, Cyclopoida and Harpacticoida orders. In addition, based on the knowledge of the role of CRGs in *Drosophila* and other terrestrial insects, we suggest potential functions of the CRGs in copepods. 

## 2. Results

### 2.1. Identification of Chemosensory Related Genes (CRGs) in Copepods

In silico mining of the NCBI Transcriptome Shotgun Assembly (TSA) database identified transcripts encoding putative CRGs in 18 different marine copepods (Table 1). The Calanoida order included the majority of the species (15/18), followed by two members of the Cyclopoida order and a single one of the Harpacticoida order (Table 1). Within the Calanoida order, most of the transcriptomes were from members of the Calanidae family (e.g., *Calanus finmarchicus*, *Calanus helgolandicus*, *Neocalanus flemingeri*, *Neocalanus cristatus*) and two from the Temoridae (*Temora stylifera*, *Temora longicornis*) family. CRGs were also identified in a single transcriptome from the Pontellidae (*Labidocera madurae*), the Pseudodiaptomidae (*Pseudodiaptomus annandalei*) and the Rhincalanidae (*Rhincalanus gigas*) families (Table 1). Almost half of the mined transcriptomes (8/18) were from adults, six from females, one from a male (*Neocalanus plumchrus*) and a single one from a mix of the two sexes (*T. longicornis*). The remaining transcriptomes were generated from mixed developmental stages or preadults CV (Table 1). Despite the differences, which can be related to dissimilarities among transcriptomes (e.g., depth of sequencing, coverage), the total number of CRGs and their distribution were comparable across all the different species investigated in this study. The number of chemosensory-related genes ranged from one to twenty-one, with the highest diversity found in *T. longicornis* (Table 1; Supplementary Table S1). 

#### 2.1.1. Ionotropic Receptors (IRs) 

**Figure 1:**
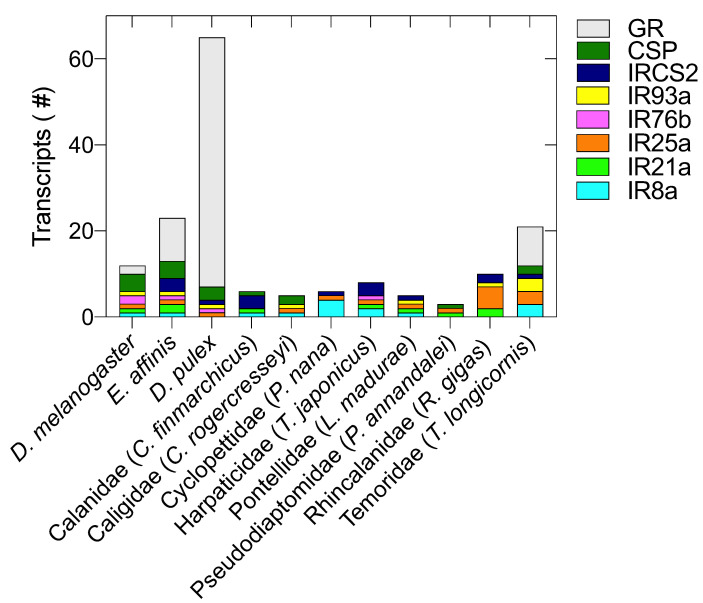
Chemosensory-related gene diversity. Distribution of transcripts encoding for chemosensory-related genes in copepods, in the insect *Drosophila melanogaster* (*D. melanogaster*) and in the cladoceran *Daphnia pulex* (*D. pulex*). CRGs include ionotropic receptors (IR8a, IR21a, IR25a, IR76b, IR93a, IRCS2), gustatory receptors (GRs), chemosensory proteins (CSPs). For the copepods examined in this study, the diversity is shown for a single member of each family (Calanidae, Caligidae, Cyclopettidae, Harpaticidae, Pontellidae, Pseudomiatomidae, Rhincalanidae, Temoridae). On x-axis, abbreviated species names: *Calanus finmarchicus* (*C. finmarchicus*), *Caligus rogercresseyi* (*C. rogercresseyi*), *Paracyclopina nana* (*P. nana*), *Tigriopus japonicus* (*T. japonicus*), *Labidocera madurae* (*L. madurae*), *Pseudodiaptomus annandalei* (*P. annandalei*), *Rhincalanus gigas* (*R. gigas*) and *Temora longicornis* (*T. longicornis*).

Transcripts encoding for conserved (IR8a, IR21a, IR25a, IR93a) and divergent (IRCSs) IRs were identified in almost all copepods, except for the co-receptor IR76b, which was exclusively found in *T. japonicus* (Figure 1; Supplementary Table S1). IR25a was the receptor mostly represented (16/18), absent only in *C. hyperboreus* and *C. finmarchicus* (Supplementary Table S1). IR8a and IR93a were found in twelve and ten species, respectively, followed by IR21a, which was present in seven species (Supplementary Table S1). Divergent IRCS2 was found in twelve copepods and showed the highest interspecies diversification in terms of number of transcripts. The majority of the identified IRs encoded for full-length proteins with both the predicted “Lig_chan” (PF00060) and “Phosphatidylethanolamine-binding protein” (PBP) (PF10613S1) domains. The number of partial proteins (positive reciprocal BLAST but no structural domains) was low, and they were found only in the IR21a (6) and IRCS2 (2) classes (Supplementary Table S2). Most of the identified IR transcripts shared the same top BLAST hit, which was the query protein from *E. affinis* (Supplementary Table S2). In contrast, for transcripts annotated as IR8a and IR25a top hits were respectively homologous from the insect *Blattella germanica* and the salmon louse *Lepeophtheirus salmonis* (Supplementary Table S2). 

#### 2.1.2. Chemosensory Proteins (CSPs), Gustatory Receptors (GRs), Odorant Receptors (ORs) and Odorant-Binding Proteins (OBPs)

Transcripts encoding for chemosensory proteins (CSPs) were identified in 9/18 copepod species. A single transcript encoding for CSP was identified in all copepods, with the exception of *T. longicornis*, which had two transcripts (Figure 1, Supplementary Table S1). The reciprocal BLAST of all transcripts resulted in *E. affinis* proteins as the top hit (although annotated as “uncharacterized protein”) and contained the typical structural domains OSD (Pfam03392) (Supplementary Table S2). The presence of transcripts encoding for gustatory receptors (GRs) was confirmed only in *T. longicornis.* In silico mining, using *E. affinis* queries, resulted in the identification of nine transcripts encoding GRs with the typical conserved “7tm_7” domain (Pfam08395). All transcripts, when reciprocal-blasted, were highly similar to *E. affinis* gustatory receptors (32 and 68 classes), although their E-values were very low (E^−04^ to E^−14^) (Supplementary Table S2). To confirm the lack of GRs in copepods, we also mined the transcriptomes using GR queries from *D. melanogaster* and *D. pulex*. These additional searches did not generate positive results. Similarly, searches of ORs and OBPs using queries from *E. affinis*, *D. melanogaster* and *D. pulex* did not generate significant results in any of the mined transcriptomes.

### 2.2. CRG Diversity and Phylogenetic Analysis 

The examined copepods showed a lower CRG number (average number of transcripts = 6) compared with *D. melanogaster* (n = 12), *E. affinis* (n = 23) and *D. pulex* (n = 65). *T. longicornis* was the copepod with the highest diversification, with a total of 21 transcripts encoding for CRGs; this number was highly comparable with *E. affinis*, which, like *T. longicornis*, is a member of the Temoridae family. *R. gigas*, with 10 total CRGs, was the closest copepod to *D. melanogaster.*

**Figure 2:**
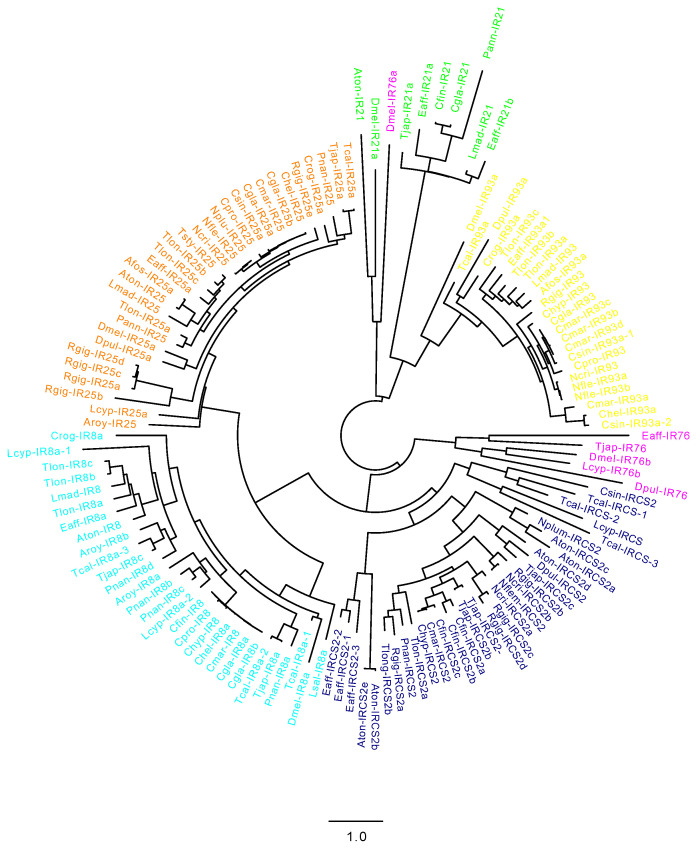
Cladogram of ionotropic receptors (IRs) identified in this study. Colors indicate the different classes. In addition to the sequences identified in this study, the analysis includes also CRGs from *D. melanogaster* and *D. pulex* and from copepods previously identified (see manuscript for details). For the analysis, amino acid sequences were aligned using ClustalW, while FAST TREE was used to build maximum-likelihood phylogenetic tree using the protein evolution model JTT + CAT. Colors are consistent with Figure 1.

In order to support the annotation of CRGs identified in this study, and to investigate the relationship with each other and with those from other species, an unrooted phylogenetic tree was generated for each class (Figure 2, Supplementary Figure S1). Figure 2 shows the unrooted tree with transcripts encoding IRs (IR8a, IR21a, IR25a, IR76b, IR93a, IRCS2) from this study, and transcripts previously identified in *D. melanogaster*, *D. pulex* and in the copepods *E. affinis*, *C. sinicus*, *A. fossae*, *L. salmonis*, *T. californicus*, *C. rogercresseyi* and *L. cyprinacea*. Our phylogenetic analysis showed a clustering pattern of the identified CRGs in agreement with their assignment; transcripts with the same annotation clustered together and with the homologs from *D. melanogaster*, *D. pulex* and *E. affinis*. IRs separated into two major clades: one with IR21a and IR93a, and the second including IR25a, IR8a, IRCS2 and IR76b (Figure 2). Within the first clade, all members of the IR21a and IR93a class clustered together based on their annotation, with an outlier within the IR21 group represented by *D. melanogaster* IR76a. In the second clade, IR8a and IR25a and IRCS2 were on the same branch, with IR76 more distant. Seventy percent of the branches were supported by bootstrap values >90% and 13% >70% (Supplementary File S2). Similarly, in the unrooted tree for CSPs and GRs, the transcripts identified in this study clustered with homologs from *D. melanogaster*, *D. pulex* and *E. affinis* (Supplementary Figure S1). For both CSP and GR analysis, more than 45% of the branches were supported by a bootstrap >90% (Supplementary File S2).

### 2.3. Relative Expression across Development and When Feeding on Toxic Diets in Calanus Finmarchicus and C. helgolandicus

**Figure 3:**
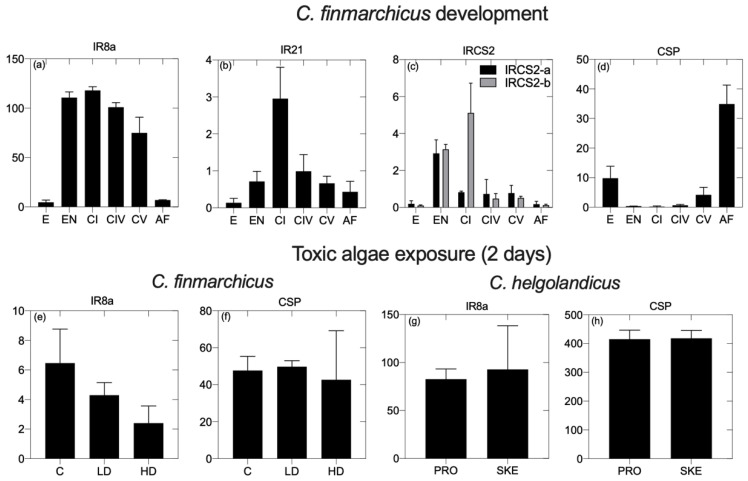
Relative expression of chemosensory genes in *Calanus finmarchicus* and *C. helgolandicus*. In the first panel (**a**–**d**), expression is shown for four ionotropic receptors (IR8a, IR21a, IRCS2) and for a single chemosensory protein (CSP) across six developmental stages: embryos (E), early nauplii (NII-NIII) (EN), early copepodids (CI), late copepodids (CIV), preadults (CV) and females (AF). Bar graphs indicate SD of the three replicates in each sample (2 replicates for CI and CIV). Second panel (**e**–**h**) shows expression for *C. finmarchicus* IR8a and CSP in females exposed for two days to the diet *R. baltica* (CONTROL; C) and two doses of *A. fundyense* (low dose [LD] and high dose [HD]). Bar graphs indicate SD of the three replicates in each sample. Relative expression of IR8a and CSP is also shown for *C. helgolandicus* females feeding on the flagellate *P. minimum* (PRO) and the oxylipin-producing *S. marinoi* (SKE). Bar graphs indicate SD of the three replicates in each sample.

Relative expression of CRGs was examined in *C. finmarchicus* across six different developmental stages (Figure 3). All transcripts encoding for IRs, which included members of the IR8a, IR21a and IRCS2 classes, showed the same pattern of expression. Relative expression was significantly lower in embryos and adults compared to all other stages (*p* < 0.05) (Figure 3a,c). For the transcript annotated as IR8a, the expression was similar between the early naupliar and the CV stages (Figure 3a). In contrast, both IR21a and transcripts encoding for IRCS2 showed a significant peak in expression in the CI stage compared with the others; in IRCS2, the expression was also high and significantly different from the others in the early naupliar stage (Figure 3b,c). By contrast, relative expression of CSP was high in embryos and adult females, and significantly lower in all other stages. Significant differences were also found between these two stages with a significantly high expression in adults (Figure 3d).

Expression of IR8a, IR93a and IRCS2 transcripts in *C. finmarchicus* females feeding on *R. baltica* and *A. fundyense* for two days was very low (RPKM<1) and did not significantly change between treatments (data not shown). The expression of IR8a and CSP was higher (RPKM<1), but similarly to the other receptors, it did not significantly change with the toxicity (Figure 3e,f). In *C. helgolandicus*, the pattern of expression was similar to the one reported for its congener, although relative expression for the examined transcript was higher (Figure 3g,h). Relative expressions of both IR8a and CSP did not change with the toxic diet being high, and was not significantly different in females feeding on *P. minimum* and *S. marinoi* (Figure 3g,h).

## 3. Discussion

Aquatic systems can be considered a landscape of smells (“smellscape”) [27], a blend of chemical cues released in the fluid that must be detected and analyzed. This scenario is made even more intricate considering the negative impact of manmade chemicals on the receptive skills of aquatic organisms [32]. Copepods possess a varied array of mechanical and chemical receptors to interact with the surrounding environment [28]. Chemical signals are used by these organisms for different purposes, including communication among conspecifics, detection of prey and evasion from predators [28,29].

The complexity of the chemosensory system, allowing an organism to sense chemical cues, has been well studied in arthropods, with most of the work on the insect *D. melanogaster* [28]. Despite various studies on crustacean chemoreception, relatively little is known about their chemosensory system at the molecular level. Recent studies have investigated chemoreceptor proteins in the decapod *Panulirus argus* [16] and in several copepods, including the brackish water *E. affinis* [2]. 

The ever-increasing use of ‘omic approaches in copepod studies [33,34] has opened the way to a deeper understanding of the transcripts encoding for chemoreceptor proteins. The overarching aim of this study has been to expand the molecular understanding of the ionotropic (IR), gustatory (GR) and odorant (OR) receptor families, of the chemosensory proteins (CSPs) and of the insect-type odorant-binding proteins (OBPs), which regulate the transport of ligands to the receptors. Out of the eighteen transcriptomes for marine copepods mined in this study, sixteen (89%) belong to Calanoida, which is not surprising, considering that species from this order are the most effective colonizers of the pelagic environment and overwhelmingly dominate the pelagic domain [35]. In spite of some differences, the distribution and diversity of CRGs is similar among the investigated copepods, with some peculiarities observed between the Calanoida, Cyclopoida and Harpacticoida families. Among these copepod families, most live in water columns (Calanidae, Rhincalanidae, Temoridae) being planktonic *par excellence*, some live near the bottom (Harpacticidae, Pseudodiaptomidae) and Caligidae are found in association with other animals as ectoparasites.

Ionotropic receptors (IRs) are considered the most ancient arthropod CRGs, dating back to the Protostomia [7]. As multimodal receptive genes, they are involved in olfactory response, taste sensation and response to environmental stimuli such as humidity and cooling temperatures [14]. IRs have been best characterized in *D. melanogaster*, which possesses 63 IRs, including the broadly expressed co-receptors and the selectively expressed tuning receptors. Functional studies of IRs are largely lacking mainly due to the limitation of genetic approaches; in *D. melanogaster*, function is known only for 18 IRs, which are mostly the ones expressed in the adult antenna [4]. Due to their nature, co-receptors are activated by different stimuli, and tuning receptors usually bind one or two co-receptors. IR25a and IR93a have been considered outside the insect clade, whereas IR21a, IR76b and IR8a were supposed as insect-specific [7] and only recently have been reported in crustaceans [2,16]. Our study not only confirms that these IRs are not “antennal” insect-specific, but it also expands the knowledge of their presence and distribution in different copepod families. IR8a, IR25a, IR76b, IR93a were found in almost all copepods with the exception of IR76b, which was only found in the neritic harpacticoid *T. japonicus*. The reason why IR76b is present only in *T. japonicus* is still unknown. We could speculate that the benthic habit could imply the detection of different stimuli, but this deserves further investigation. In *D. melanogaster*, IR76b is mostly involved in taste detection and is activated by several stimuli such as amino acids, calcium, pyrrolidine and phenylethylamine [4]. The expression of IRs has been reported to be sex-biased, with a high level in males. Compared with females, in *E. affinis* and *Oithona nana* males, high expression was reported, respectively, for IR8, IR25 and IR76 and for two “ionotropic glutamate receptor subunits“ [2,36]. In both studies, it is suggested that these receptors might have a specific role in mating. With the exception of one transcriptome, all resources mined in this study have been generated from mixed developmental stages or females; thus, we could not test this hypothesis. 

Consistently with an ancestral role and more conserved functions, IR25a is the most represented co-receptor, found in 16/18 copepods. This receptor is involved in gustation and hygrosensation (moist and dry) and is expressed in *D. melanogaster* larvae and adults [4]. *C. helgolandicus* relative expression of IR25a was high in females fed on the dinoflagellate *P. minimum* or the oxylipin-producing diatom *S. marinoi* over two days (data not shown). The second most abundant receptor is IR8a (12/18), followed by IR93a (10/12) and IR21a (12/18). IR8a is involved predominantly in olfaction and has been reported in many crustaceans such as *Homarus americanus*, spiny lobsters, shrimps and copepods [2,16]. Eyun et al. [2] suggested that IR8a evolved first in the pancrustaceans and was secondarily lost in branchiopods. In *D. melanogaster*, IR8a was expressed in adult flies, while in *C. finmarchicus*, this receptor showed a significantly low expression in embryos and females compared with nauplii and copepodids. This could suggest that in copepods, IR8a may have an additional role during development. In both *C. finmarchicus* and *C. helgolandicus*, IR8a was also expressed in females incubated with food for two days, and no effect was found when one of the two algae was toxic. The tuning receptor IR21a is present only in 7/18 copepods; this receptor has been previously reported in other copepods (*Caligus rogercresseyi* and *E. affinis*) and hexapods (insects) [2]. IR21a is involved in thermosensation, being activated by cool temperatures. In *C. finmarchicus*, the expression of this receptor was significantly high in the CI developmental stage. In *D. melanogaster*, it is expressed in adults and in larvae [4]. Overall, based on the insect function, some IRs could also mediate in copepod olfactory signaling; however, little is known still on the functional roles of IRs, and much more remains to be discovered. 

In crustaceans, GRs have rarely been identified and their anatomical location or involvement in chemical sensing has not been demonstrated. *D. pulex* is the crustacean with the highest diversity (58 GRs), but only few GRs have been found in some species of Copepoda and in a barnacle (Cirripedia) [2,37]. Numerous works demonstrate the presence of chemoreceptors on copepod mouthparts [38,39,40]. These sensors may be used for the direct selection (also including rejection) of food particles [41] and may trigger the handling of the item [42]. Chemoreceptors are also present along the long first antennae of copepods, likely being involved in the perception of chemical signals from the far field [41]. This evidence seems in contrast with the absence of GR genes in all 17 transcriptomes mined in the present investigation, with the only exception of *T. longicornis*. Eyun et al. [2] concluded that GRs appeared early in metazoan evolution but expanded only in some arthropod groups, which included Insecta and some Chelicerata, but not most Crustacea. Another possible explanation could be linked to the documented involvement of *Drosophila* GRs in the perception of stimuli beyond peripheral nonvolatile chemicals, such as CO_2_ and light [43]. It might be likely that copepod GR genes may be used by selected species only (in this case, *T. longicornis*), not representing a universal common trait. On the other hand, gustatory functions in copepods may be associated with other gene families. To solve this issue, more specifically focused experiments are needed, exposing the animals to known chemical signals and analyzing the associated transcriptome.

Odorant receptors (ORs) constitute an expanded lineage within the GR superfamily, although to date it has been reported that they are restricted to insects [43]. The evolution of ORs has been hypothesized to be associated with the insect’s colonization of land enabling the detection of volatile compounds in air [36,44,45]. Unsurprisingly, OR family has been reported absent in crustaceans, including the water flea *D. pulex* and the copepod *E. affinis* [2]. Here, the mining of 18 transcriptomes from copepods belonging to different orders confirmed the lack of these proteins. It has to be noticed that automated annotation of the *T. stylifera* transcriptome (used also in this study) reported several transcripts annotated as putative OBPs [46]. The discrepancies between our results and those reported by Russo et al. [46] could be explained by the more stringent searching criteria (exclusion of partial proteins) and the different protein database (Pfam vs. Interpro) used in this study. However, this result supports the need to integrate automatic software annotation of transcriptome sequences with more in-depth manual analysis. The lack of OBPs and ORs is consistent with previous studies, supporting the conclusion that both are specific to insects and absent in other arthropods. This outcome stimulates some reflection on such an absence. In the copepod literature, several works specifically report on the odor perception of different species [25,39,47]; thus, the lack of both ORs and OBPs may seem counterintuitive. Odorants typically refer to volatile compounds perceivable through olfaction, replaced by waterborne signals in the water medium [48]; however, some marine organisms do present OR genes. On these grounds, Mollo et al. [48] thus propose that small, water-insoluble molecules may act as contact odorants, with a “reversal of senses” between aquatic and air environments (see their Figure 1). Due to the multiplicity of roles, it is likely that in those organisms, the odor perception is accomplished by IRs, which need a stronger and/or very near stimuli source compared to ORs. Overall, these findings underline the importance of further investigating this specific issue, which at present is still unclear not only for copepods, but in general for aquatic animals. 

## 4. Materials and Methods

### 4.1. In Silico Mining, Reciprocal BLAST and Protein Domain Identification 

The presence of gustatory receptors (GRs), odorant receptors (OR), ionotropic receptors (IRs), chemosensory proteins (CSPs) and insect-type odorant-binding proteins (OBPs) was examined in copepods. Among the IRs, we searched for IR8a, IR25a, IR76b, IR93a (coreceptors), IR21a and the divergent IRCS2. In silico searches for putative transcripts encoding these receptors and proteins were performed using a well-established vetting protocol that involves mining, a reciprocal BLAST and a protein structural motif analysis step [40,41,42,43,44,45,46,47,48,49,50,51]. The Transcriptome Shotgun Assembly (TSA) database on the National Center for Biotechnology Information (NCBI) was mined (July 2022) using query sequences from the copepod *E. affinis* to search for GRs, IRs and CSPs, setting the limit to Copepoda (Taxid: 6830). Additional searches were performed for odorant receptors (ORs) and odorant-binding proteins (OBPs) (absent in *E. affinis*), and for GRs using protein queries from *D. melanogaster.* Resulting transcripts from all searches were reciprocal-blasted to confirm their identity. Briefly, each putative transcript was fully translated using ExPASy [52], and then the deduced protein was used to query the NCBI nonredundant (nr) protein database (blastp algorithm). Pfam software [53] was used to assess the presence of the expected protein structural motif. IRs have several transmembrane domains: an extracellular ligand binding domain (LBD) consisting of two half-domains (S1 and S2), to which L-glutamate, glycine or serine agonists bind; and a ion channel domain (ICD) forming a ion channel, consisting of three transmembrane domains (M1, M2, M3) and a pore loop (P). Based on Pfam, predicted IRs had to include a “Lig_chan” domain (PF00060) (which contains M1, P, M2, S2, and M3) and the “Phosphatidylethanolamine-binding protein” (PBP) domain (PF10613S1) which includes the S1 of the LBD. For GR receptors, Pfam predicted the presence of the “7tm chemosensory receptor “ (Pfam08395), while for CSPs, the presence of the “Insect pheromone-binding” (OS-D) domain (Pfam03392) was predicted. Only transcripts encoding proteins that included the expected domains were considered for downstream analyses. 

### 4.2. Cladogram of Copepod Chemosensory-Related Genes

A phylogenetic analysis using sequences from this study (Table 1) from other copepods (*E. affinis, C. rogercressey*, *L. cyprinacea, L. salmonis, T. californicus*) from *D. melanogaster* and *D. pulex* [2] was used to support the assignment of the predicted chemosensory-related genes in this study. An unrooted phylogenetic tree was generated using amino acid sequences from all species that were aligned using ClustalW software (Galaxy version 2.1) [54], while FASTTREE was used to build a maximum-likelihood phylogenetic tree (Galaxy Version 2.1.10+galaxy1) using the protein evolution model JTT+ CAT [55]. For the sequences identified in this study, we only included in the analysis transcripts encoding for full-length proteins with the expected structural motifs. 

### 4.3. Relative Expression of Chemosensory Related Genes in Calanus Finmarchicus and C. helgolandicus across Development and When Exposed to Toxic Algae

Relative expression of chemosensory-related genes was examined in the copepods *C. finmarchicus* and *C. helgolandicus* using previously published RNASeq data [56,57,58]. The expression data for CRGs obtained from the datasets were normalized using the reads per kilobase per million mapped reads (RPKM) method [59]. A 2-way ANOVA (*p* < 0.05) followed by post hoc Tukey’s test was used to assess statistical significance in each study. In *C. finmarchicus*, the expression of CRGs was examined across six different developmental stages and when exposed to a toxic diet. Developmental expression included six stages: embryos, early nauplii, early copepodids (CI), late copepodids (CIV), preadults (CV) and females. Each stage included three samples processed for RNA-Seq (exception CI and CIV with two replicates), and the expression rate was measured by mapping each library against the *C. finmarchicus* reference transcriptome (NCBI: PRJNA236528) using bowtie software (v.2.0.6). The second dataset included *C. finmarchicus* females incubated for two and five days with three experimental diets: control (*Rhodomonas* sp.) and two doses (low and high) of the saxitoxin-producing dinoflagellate *Alexandrium fundyense*. Females were exposed to the three diets, and after two days, samples were harvested for RNA-Seq (three replicates/treatment). Expression was quantified by mapping each RNA-Seq library against the *C. finmarchicus* reference transcriptome (NCBI: PRJNA236528) using bowtie software (v.2.0.6). 

For *C. helgolandicus*, CRG expression was examined in laboratory-incubated females feeding for five days on the oxylipin-producing diatom *Skeletonema marinoi* and the control diet *Prorocentrum minimum*. In brief, *C. helgolandicus* females were fed for five days with either *S. marinoi* or *P. minimum* at 1 mg CL^−1^ (three replicates each). RNA-Seq libraries were pooled to generate a de novo assembly (NCBI: PRJNA640515) used to quantify expression levels by self-mapping using bowtie software. 

## 5. Conclusions

Genomic and transcriptomic approaches provide unique opportunities to investigate the molecular-level mechanisms in chemical signal perception. This study opens new perspectives on the investigation of specific copepod genes that can be used as biomarkers in response to environmental triggers, such as chemical mediators released by individuals of the same or other species, or present in the environment as pollutants. The limited availability of data on crustaceans in general [15], and on copepods in particular (this study), presently allow for the depiction of an initial framework. More extensive transcriptomics analyses [15], together with the creation of chemical compound libraries [27], are advocated in order to gain an overall view of the processes regulating chemical communication in aquatic environments. Focused investigation on selected developmental stages and sexes, in tandem with the identification of CRGs in selected body parts (e.g., cephalic area, mouthparts, genital segment), will additionally clarify the ontogenetic development and regionalization of chemical perception.

## Figures and Tables

**Table 1:** List of copepod transcriptomes mined for chemosensory-related genes. Transcriptomes were publicly available through the transcriptome shotgun assembly (TSA) database on the National Center for Biotechnology Information (NCBI). For each transcriptome, genus, species, NCBI Bioproject number and species developmental stages are listed.

Genus	Species	Bioproject	Developmental stages
*Acartia*	*tonsa*	PRJEB20069	mix stages (embryo, nauplii, copepodids, preadult, adult)
*Calanus*	*finmarchicus*	PRJNA236528	mix stages (embryo, nauplii, copepodids, females)
*Calanus*	*glacialis*	PRJNA237014	females
*Calanus*	*helgolandicus*	PRJNA640515	females
*Calanus*	*hyperboreous*	PRJNA744376	females
*Calanus*	*marshallae*	PRJNA745090/PRJNA662858	preadult (CV)
*Calanus*	*propinquous*	PRJNA669816	females
*Labidocera*	*madurae*	PRJNA324849	mix copepodids (CIII-CV), females
*Neocalanus*	*cristatus*	PRJNA662858	preadult (CV)
*Neocalanus*	*flemingeri*	PRJNA324453	females
*Neocalanus*	*plumchrus*	PRJNA662858	male
*Pseudodiaptomus*	*annandalei*	PRJNA558682	embryos, nauplii, copepodids, females, males
*Rhincalanus*	*gigas*	PRJNA666170	preadult (CV), adult
*Temora*	*longicornis*	PRJNA577564	males and females
*Temora*	*stylifera*	PRJNA632714	females
*Apocyclops*	*royi*	PRJEB28764	not indicated NCBI
*Paracyclopina*	*nana*	PRJNA268783	not indicated NCBI
*Tigriopus*	*japonicus*	PRJNA274317	not indicated NCBI

## Data Availability

The National Center for Biotechnology Information (NCBI) Bioproject numbers for the datasets examined in the present study are indicated in Table 1. Supplementary File S1 includes FASTA files for the transcript encoding protein identified in this study. Supplementary File S2 includes bootstrap values (red) for the unrooted cladograms generated for IRs (Figure 2), CSPs (Figure S1a) and GR (Figure S1b).

## Supplementary Materials

The following supporting information can be downloaded at: https://www.mdpi.com/article/10.3390/md20110681/s1, Table S1: Summary of transcripts encoding for chemosensory genes identified in several copepods. The list includes ionotropic receptors (IRs), gustatory receptors (GRs), chemosensory proteins (CSPs). IR number includes the different classes (IR8a, IR21a, IR25a, IR76b, IR93a, IRCS2) including only transcripts that passed the reciprocal BLAST step and showed the predicted Pfam domain.; Table S2: Summary of reciprocal BLAST results for the investigated IRs, CSP and GRs. For each species, reciprocal BLAST includes species, NCBI accession number, E value, annotation result and top hit (species). Additionally, information on presence of Pfam domains and the completeness of the predicted protein (Partial/Full). Figure S1: Cladogram of chemosensory proteins (CSPs) [a] and gustatory receptors (GRs) [b] identified in this study. The analysis also includes transcripts from *Drosophila melanogaster* and *Daphnia pulex* and from copepods previously identified (see manuscript for details). For the analysis, amino acid sequences were aligned using ClustalW, while FAST TREE was used to build maximum-likelihood phylogenetic tree using the protein evolution model JTT + CAT.
