# Supplementary material for: Chemosensory-Related Genes in Marine Copepods
Source: Mar Drugs. 2022 Oct 29;20(11):681. doi: 10.3390/md20110681 (PMC9692914; doi:10.3390/md20110681)
Supplement: Supplementary file 1 [file marinedrugs-20-00681-s001.zip › marinedrugs-1973025-supplementary/Supplementary/Roncalli-CRG-TableS1.pdf]

Table S1: Summary of transcripts encoding for chemosensory genes identified in several copepods. The list includes ionotropic receptors (IRs), gustatory receptors (GRs), chemosensory proteins (CSPs). IR number includes the different classes (IR8a, IR21a, IR25a, IR76b, IR93a, IRCS2) including only transcripts that passed the reciprocal BLAST step and showed the predicted Pfam domain.

		**Ionotropic receptors (IR)**						**CSP**	**GR**
		**IR8a**	**IR21a**	**IR25a**	**IR76b**	**IR93a**	**IRCS2**		
A. tonsa	Acartiidae	1	1	1	0	0	5	1	0
C. finmarchicus	Calanidae	1	1	0	0	1	3	1	0
C. glacialis	Calanidae	2	1	2	0	1	0	0	0
C. helgolandicus	Calanidae	1	0	1	0	1	0	1	0
C. hyperboreous	Calanidae	1	0	0	0	1	1	0	0
C. marshallae	Calanidae	1	0	1	0	4	1	0	0
C. propinqus	Calanidae	1	0	1	0	1	0	1	0
L. madurae	Pontellidae	1	1	1	0	1	1	0	0
N. cristatus	Calanidae	0	0	1	0	1	2	1	0
N. flemingeri	Calanidae	0	0	1	0	2	1	1	0
N. plumchrus	Calanidae	0	0	1	0	0	2	1	0
P. annandalei	Pseudodiaptomidae	0	1	1	0	0	0	1	0
R. gigas	Rhincalanidae	0	2	5	0	1	2	0	0
T. longicornis	Temoridae	3	0	3	0	3	1	2	9
T. stylifera	Temoridae	0	0	1	0	0	0	0	0
A. royi	Cyclopidae	2	0	1	0	0	0	0	0
P. nana	Cyclopoida	4	0	1	0	0	1	0	0
T. japonicus	Harpacticidae	2	1	1	1	0	3	0	0
