# Supplementary figures and images for: Chemosensory-Related Genes in Marine Copepods
Source: Mar Drugs. 2022 Oct 29;20(11):681. doi: 10.3390/md20110681 (PMC9692914; doi:10.3390/md20110681)

A

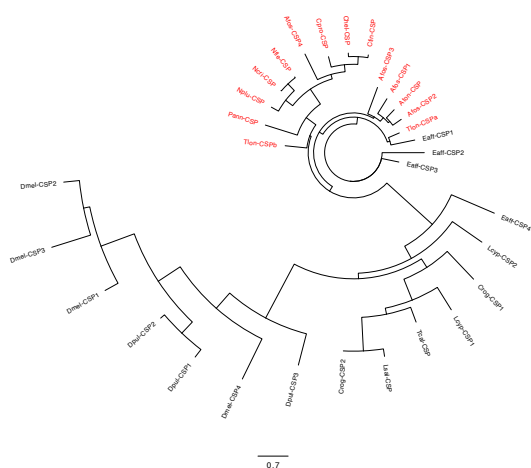

B

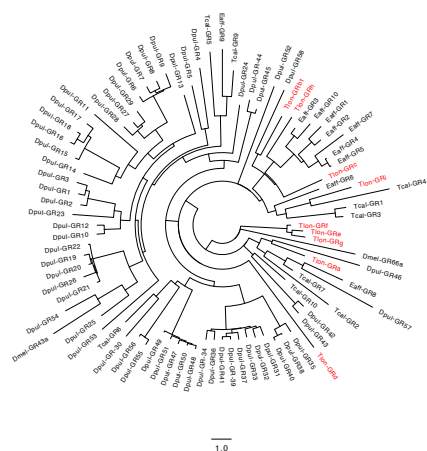

Supplement: Supplementary file 1 [file marinedrugs-20-00681-s001.zip › marinedrugs-1973025-supplementary/Supplementary/Roncall-CRG-FigureS1.pdf]

## Slide 1
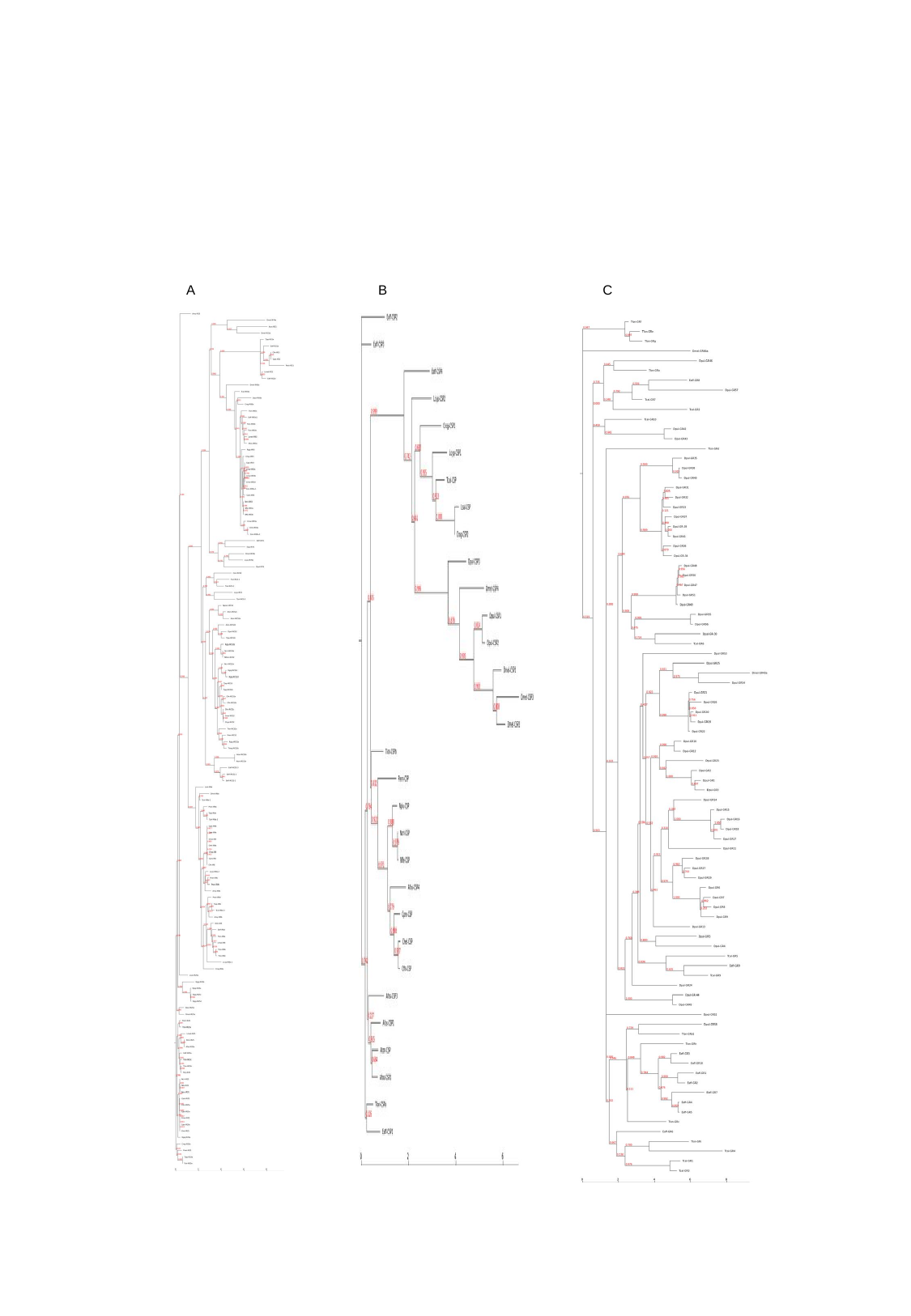

A
B
C

Supplement: Supplementary file 1 [file marinedrugs-20-00681-s001.zip › marinedrugs-1973025-supplementary/Supplementary/Supplementary File S2.pptx]
